# Age- and Sex-Related Normative Anterior Segment Parameters Using Swept-Source OCT: Insights from Pediatric to Elderly Populations

**DOI:** 10.3390/jcm14217558

**Published:** 2025-10-24

**Authors:** Hatice Kubra Sonmez, Zeynep Akkul, Hidayet Sener, Erinc Buyukpatır Deneme, Elif Er Arslantas, Cem Evereklioglu, Fatih Horozoglu, Osman Ahmet Polat, Hatice Arda

**Affiliations:** 1 Department of Ophthalmology, Medical Faculty, Erciyes University, Kayseri 38039, Türkiyefhoroz@yahoo.com (F.H.); haticearda75@gmail.com (H.A.); 2 Department of Ophthalmology, Bursa Yuksek Ihtisas Training and Research Hospital, Bursa 16310, Türkiye; drzeynepakkul@gmail.com; 3 Department of Ophthalmology, Develi Dr. Ekrem Karakaya State Hospital, Kayseri 38400, Türkiye; erincbuyukpatir@gmail.com; 4 Department of Ophthalmology, Viransehir State Hospital, Sanlıurfa 63700, Türkiye; 5 Department of Ophthalmology, Maya Eye Hospital, Kayseri 38230, Türkiye

**Keywords:** keratometry, cornea, anterior segment, swept-source optical coherence tomography, Anterion

## Abstract

**Objectives**: To establish normative data for anterior segment parameters in healthy pediatric and adult populations using swept-source optical coherence tomography (SS-OCT), and to evaluate the influence of age and sex on these parameters. **Methods**: This retrospective study included the right eyes of 390 healthy participants. Subjects were divided into three age groups: Group 1 (6–17 years, *n* = 97), Group 2 (18–45 years, *n* = 144), and Group 3 (46–77 years, *n* = 149). All patients were categorized according to their biological sex as female and male. Exclusion criteria were corneal pathology, prior intraocular/refractive surgery, recent contact lens use, severe dry eye, ectatic disorders, low-quality imaging, and refractive error of ±2.0 D or greater. Measurements of anterior and posterior keratometry, total corneal power (TCP), central corneal thickness (CCT), thinnest corneal thickness (TCT), pupil diameter (PD), lens thickness (LT), and white-to-white distance (WTW) were obtained using the Anterion^®^ SS-OCT system. Data were analyzed using SPSS software. **Results**: Group 1 demonstrated the highest PD and CCT values, whereas LT was lowest. In adults, LT increased with age and was significantly higher in males older than 45 years. Keratometric analysis revealed greater anterior and total steep astigmatism in the pediatric group, independent of sex. Adult females had significantly higher anterior and posterior keratometry values compared with males. In the pediatric cohort, females exhibited greater CCT, while WTW varied with age. PD decreased with age, whereas LT increased. **Conclusions**: Anterior segment parameters measured with SS-OCT show significant variations across different age groups and between sexes. Normative data, particularly for pediatric and adult populations, may serve as valuable reference values in keratorefractive surgical planning and corneal pathology assessment. Future studies with larger cohorts, especially in pediatric populations, are warranted.

## 1. Introduction

Anterior segment analysis plays a critical role in the diagnosis of corneal diseases and in preoperative evaluation prior to various surgical interventions, including cataract surgery, corneal transplantation, and laser corneal refractive procedures. Devices such as Scheimpflug cameras, Scheimpflug–Placido topographers, optical coherence tomography (OCT), and color light-emitting diode (LED) corneal topographers enable the measurement of multiple parameters, including anterior and posterior corneal curvature, central corneal thickness (CCT), total corneal power (TCP), white-to-white distance (WTW), anterior chamber depth, pupil diameter (PD), lens thickness (LT), and corneal wavefront analysis [1,2,3].

Keratometry is one of the fundamental measurements in anterior segment evaluation and can be obtained with these devices. Studies have demonstrated that the use of total keratometry in intraocular lens (IOL) power calculation provides more accurate outcomes compared with conventional keratometry, which is based solely on anterior corneal curvature [4,5]. Furthermore, posterior corneal astigmatism has been shown to be a critical parameter, particularly in toric IOL selection, as it may improve the refractive outcomes of cataract surgery [6,7,8,9]. Therefore, precise measurement of corneal refractive power and its integration into clinical practice are of great importance for surgical success.

With advances in cataract and refractive surgical techniques, the accurate measurement of corneal power and pupil size has become increasingly important [10]. Parameters such as CCT, mean keratometry (Kmean), flat keratometry (Kflat), steep keratometry (Ksteep), and PD are key determinants in procedures including excimer laser ablation and multifocal IOL (MIOL) implantation [11,12]. New imaging technologies have facilitated more detailed visualization of anterior segment structures, offering significant advantages in both pre-refractive surgical screening and diagnostic evaluation [13]. In particular, anterior and posterior corneal analysis, elevation maps, and detailed corneal imaging are valuable for the assessment of ectatic disorders, corneal astigmatism, and refractive surgical planning.

Projection-based systems remain the most commonly used tools for assessing the anterior corneal surface; however, they have significant limitations in evaluating posterior corneal curvature. In contrast, slit-scanning elevation, Scheimpflug imaging, and OCT can assess both anterior and posterior corneal surfaces. Cross-sectional images obtained with these technologies assist surgeons in optimizing IOL power calculations, particularly in cases requiring premium IOLs. Noninvasive imaging modalities that allow full corneal visualization are increasingly being integrated into clinical practice [14]. OCT has been reported to provide highly reproducible measurements of anterior and posterior corneal parameters in normal eyes, post-keratoplasty patients, and keratoconus cases, and it is now widely used in clinical applications [15,16].

More recently, swept-source OCT (SS-OCT) technology has enabled deeper and higher-contrast imaging of the entire anterior segment [17]. The Anterion^®^ system (Heidelberg Engineering, Germany), a high-resolution anterior segment imaging device based on SS-OCT, has recently become available in clinical practice [18]. This multimodal device allows both axial length measurement for ocular biometry and detailed anterior segment imaging. Its “cornea display” module provides multiple parameters, including anterior and posterior keratometry, PD, CCT, and elevation maps.

It is well recognized that anterior segment parameters vary in the pediatric population. Studies focusing on IOL implantation or pediatric keratoconus have highlighted that understanding age-related changes in these parameters may help reduce refractive errors in clinical outcomes. Nevertheless, further research is warranted in this field [19,20].

The aim of this study was to establish normative data for anterior segment parameters in healthy pediatric and adult populations using SS-OCT and to provide a reference framework for future keratorefractive and anterior segment research.

## 2. Materials and Methods

### 2.1. Study Design and Patient Selection

This retrospective observational cohort study was approved by the Ethics Committee of Erciyes University Faculty of Medicine (Approval No: 2024/265) and conducted in accordance with the tenets of the Declaration of Helsinki. A total of 390 right eyes from 390 healthy individuals were included. All participants underwent a comprehensive ophthalmological examination. Exclusion criteria were corneal abnormalities, history of intraocular or refractive surgery, recent contact lens wear, corneal pathology, severe ocular surface dryness, ectatic disorders, poor-quality scans, and refractive error of ±2.0 D or greater. Participants were categorized into three age groups: Group 1 (6–17 years), Group 2 (18–45 years), and Group 3 (46–77 years). In the study, all patients were categorized according to their biological sex as female or male.

### 2.2. Imaging Protocol

During imaging, participants were instructed to fixate on the central target of the device. Only high-quality scans with a “green” quality code were included in the analysis. Imaging was performed under mesopic conditions using the “Cornea” and “Metrics” modes of the Anterion^®^ system (Heidelberg Engineering, Heidelberg, Germany; software versions: HX 2.5.9.2048, VWM 1.4.4.0, AQM 1.4.4.0). The Anterion^®^ employs swept-source OCT (SS-OCT) technology, offering high axial and lateral resolution with a scanning speed of 50,000 A-scans per second. Using a 1300-nm light source, it provides scans up to 14.5 mm in depth and 16.5 mm in width. Corneal maps generated from SS-OCT data cover an 8-mm diameter (18). Measurements were acquired with reference to the corneal apex, aided by the device’s eye-tracking system. Images taken by the same technician were used.

### 2.3. Data Analysis

The evaluated parameters included: Anterior axial curvature parameters; mean anterior SimK, flat SimK, steep SimK, anterior steep astigmatism, posterior axial curvature parameters; mean posterior K (postK), flat posterior K, steep posterior K, posterior steep astigmatism, ray-traced total corneal power parameters; mean TCP (TCPmean), flat TCP (TCPf), steep TCP (TCPs), total steep astigmatism, pachymetric parameters; CCT, thinnest corneal thickness (TCT), other parameters; PD, WTW distance, LT. All data were exported from the Anterion^®^ system in PDF format for subsequent analysis.

### 2.4. Study Endpoints

The primary endpoint of this study was to establish normative data by determining the variations in keratometric and selected anterior segment parameters across different age groups, including the pediatric population, thereby providing guidance for future research. The secondary endpoint was to evaluate the sex-related differences within age groups, the age-related variations between sexes, and the potential effects of these variations.

### 2.5. Statistical Analysis

Statistical analysis was performed using IBM SPSS Statistics for Windows, version 22.0 (IBM Corp., Armonk, NY, USA). Normality of distribution was assessed using the Shapiro–Wilk test. Normally distributed variables were expressed as the mean ± standard deviation, while non-normally distributed variables were presented as median (1st–3rd quartile). For comparisons among the three groups, one-way ANOVA was used for normally distributed variables, and the Kruskal–Wallis test for non-normally distributed variables. For pairwise comparisons, an independent-samples *t*-test was applied to normally distributed variables and the Mann–Whitney U test to non-normally distributed variables, with Bonferroni correction. The sample size was estimated as 207 participants in total (69 subjects per group) based on a power of 0.9 (β = 0.1), an alpha level of 0.05, and an effect size of 0.25. Post hoc tests included Tukey’s HSD or Tamhane’s T2, depending on the homogeneity of variances. Effect sizes were calculated as η^2^ or Cohen’s d→r for ANOVA, and Rosenthal’s r for the Kruskal–Wallis test. Post hoc analyses of significant parameters were performed with Bonferroni correction. A *p*-value < 0.05 was considered statistically significant.

## 3. Results

### 3.1. Demographic Findings

Of the 390 eyes included in the study, 97 were in Group 1, 144 in Group 2, and 149 in Group 3. The female/male ratios in the groups were 63/34 in Group 1, 102/42 in Group 2, and 102/47 in Group 3, with no significant difference among the groups (*p* = 0.63). The mean ages were 13.47 ± 3.16, 33.29 ± 8.89, and 55.98 ± 7.33 years for Group 1, Group 2, and Group 3, respectively (mean ± SD).

### 3.2. Comparison of Age Groups

The comparative evaluation of keratometry, pachymetry, PD, LT, and corneal WTW measurements among the three groups is presented in Table 1.

When Group 1 and Group 2 were compared, CCT (*p* = 0.017, r = 0.33), TCT (*p* = 0.02, r = 0.32), and PD (*p* < 0.001, r = 0.32) were significantly higher in Group 1, while LT (*p* < 0.001, r = 0.521) was higher in Group 2. When Group 2 and Group 3 were compared; TCT (*p* = 0.001, r = 0.62), WTW distance (*p* = 0.015, r = 0.167), posterior steep astigmatism (*p* = 0.023, r = 0.151), and total steep astigmatism (*p* = 0.024, r = 0.150) were significantly higher in Group 2, while LT (*p* < 0.001, r = 0.616) was higher in Group 3. When Group 1 and Group 3 were compared; anterior steep astigmatism (*p* = 0.026, r = 0.265), total steep astigmatism (*p* = 0.022, r = 0.178), CCT (*p* = 0.001, r = 0.67), PD (*p* < 0.001, r = 0.617), and WTW distance (*p* < 0.001, r = 0.269) were significantly higher in Group 1, whereas LT (*p* < 0.001, r = 0.798) was significantly higher in Group 3. Effect size comparisons of anterior segment parameters across age groups are presented in Table 2. Accordingly, the highest effect sizes were observed in PD and LT parameters, which showed significant variability with age.

When Group 1 was subdivided into pre-adolescent and adolescent subgroups [21], no significant differences were observed in any parameter, as shown in Table 3.

### 3.3. Sex Comparison Within Groups

Intragroup comparisons based on sex revealed that in females, Sim K (*p* = 0.034, *p* = 0.031, *p* = 0.002 for Group 1, 2, and 3, respectively), posterior K flat (*p* = 0.045, *p* = 0.003, *p* = 0.004), and TCPf (*p* = 0.036, *p* = 0.018, *p* = 0.002) values were significantly higher in all three groups. Sim K flat, post K, and posterior K steep values were significantly higher in females in Group 2 (*p* = 0.012, *p* = 0.007, *p* = 0.032, respectively) and Group 3 (*p* = 0.007, *p* = 0.007, *p* = 0.036, respectively). WTW distance was significantly lower in females in Group 1 (*p* = 0.036) and Group 3 (*p* < 0.001). Conversely, TCP mean was higher in females in the same groups (*p* = 0.042, *p* = 0.008, respectively). Only in Group 3, PD (*p* = 0.004) and Sim K steep (*p* = 0.033) were higher in females, whereas LT was higher in males (*p* = 0.044). Although post K max was higher in females across all groups, the difference was statistically significant only in Group 2 (*p* = 0.036) (Table 4).

### 3.4. Age Comparison by Sex

In males, intergroup comparisons showed that anterior steep astigmatism differed significantly only between Group 1 and Group 3, being higher in Group 1 (*p* = 0.026). PD showed significant differences across all groups, being highest in Group 1 and lowest in Group 3. LT also differed significantly among all groups, being highest in Group 3 and lowest in Group 1.

In females, anterior steep astigmatism differed significantly in the comparisons between Group 2–3 and Group 1–3, with the lowest value in Group 3. Posterior and total steep astigmatism were significantly higher in Group 2 compared to Group 3 (*p* = 0.019, *p* = 0.028, respectively). CCT and TCT values were higher in Group 1 compared to Group 3, and the difference was significant (*p* = 0.001, *p* < 0.001, respectively). PD was highest in Group 1 and lowest in Group 3, whereas LT was the highest in Group 3 and the lowest in Group 1, with significant differences across all groups. WTW distance was similar between Group 1 and Group 2, while it showed significant differences between other groups, with the lowest value in Group 3. Comparisons of anterior segment parameters by sex across age groups are presented in Table 5.

In males, significant differences among groups were observed in anterior steep astigmatism (*p* = 0.021, r = 0.23), PD (*p* < 0.001, r = 0.55), and LT (*p* < 0.001, r = 0.60), with small-to-moderate effect sizes for anterior steep astigmatism and large effect sizes for PD and LT. In females, anterior steep astigmatism (*p* = 0.001, r = 0.28), posterior steep astigmatism (*p* = 0.021, r = 0.20), TCP astigmatism (*p* = 0.014, r = 0.21), PD (*p* < 0.001, r = 0.52), WTW distance (*p* < 0.001, r = 0.29), and LT (*p* < 0.001, r = 0.61) differed significantly among groups, with large effect sizes for PD and LT, and small-to-moderate effect sizes for the other parameters. CCT and TCT parameters in females showed significant differences among groups but with very low effect sizes (CCT: *p* = 0.001, η^2^ = 0.064; TCT: *p* < 0.001, η^2^ = 0.053). In post-hoc analyses (Bonferroni corrected), in males, anterior steep astigmatism was significantly different between Group 1 and Group 3, while PD and LT differed among all group pairs. In females, anterior steep astigmatism was significantly different between Group 3 and Groups 1 and 2, posterior steep astigmatism between Groups 2 and 3, and TCP astigmatism between Groups 2 and 3. PD and LT differed among all group pairs, while WTW distance differed significantly between Groups 1–3 and Groups 2–3.

## 4. Discussion

The primary outcome of this study was to provide normative anterior segment data by age groups, obtained from quantitative measurements with anterior segment SS-OCT. The secondary outcome was to evaluate the effects of age groups and sex on these anterior segment parameters. When the obtained data were evaluated, pupil diameter was found to be higher in the pediatric age group (Group 1), both overall and when males and females were analyzed separately. However, when sex groups were compared, a higher pupil diameter was observed only in females over 45 years of age (Group 3). Kiel et al. [22], in their study including 18,335 eyes, reported the median mesopic pupil size in the right eye as 4.19 mm in the 40–80 age group and showed that pupil diameter decreases with age. They also reported that pupil diameter was wider in females. Winn et al. found in 91 healthy eyes that pupil size was independent of sex but decreased with age [23]. On the other hand, a study including 30 eyes reported no relationship between pupil size and age [24]. A large cohort study conducted in the pediatric population reported a mean mesopic pupil size of 5.64 mm in healthy eyes aged 2.5–18 years [19]. Consistent with the literature, our study also demonstrated larger mesopic pupil diameters in the pediatric age group, while sex did not significantly affect pupil size in adults under 45 years. However, since most publications in the literature focus on adult groups, there is a need for more studies on pediatric populations.

In our study, lens thickness was found to increase with age, being higher in males over 45 years, whereas no significant difference between sexes was detected in pediatric and adult groups under 45 years. Lu et al., in a study of 596 children aged 6–16 years, reported a median lens thickness of approximately 3.30 mm and found it to be thicker in females [20]. In our pediatric group (6–17 years), the mean lens thickness was 3.49 mm and, although higher in females, the difference was not statistically significant. In a population-based study of healthy adults, the mean lens thickness measured sonographically was 3.95 mm and was reported to increase in males and with advancing age [25]. Similarly, a study using SS-OCT, including 76 participants aged 18–86 years, demonstrated a positive correlation between age and lens thickness [26]. Another study reported that lens thickness increased with age but was not influenced by sex [27]. These findings may be related to age-associated degenerative changes and structural differences in the capsule. Our results are largely consistent with the literature.

Regarding keratometric analyses, anterior and total steep astigmatism were higher in the pediatric group, with no significant association with sex. No significant relationship was observed between age and other keratometric parameters. In adult groups, anterior keratometric parameters, including mean anterior Sim K, Sim K flat, Sim K steep, TCP mean, TCP flat, and posterior keratometric parameters, including mean posterior K, posterior K flat, posterior K steep, and posterior K max were significantly higher in females. In a study by Namba et al., anterior keratometric values (K flat, K steep, and K max) were found to be higher in females, while posterior keratometric values were higher in males [27]. However, many studies have reported steeper anterior and posterior corneal curvature in adult females [28,29,30]. Our findings largely overlap with these reports. Ethnic differences may also influence these parameters. Studies conducted in different ethnicities (Caucasian, Brazilian, Korean, etc.) on anterior and posterior astigmatism yielded results comparable to ours [29,31,32,33].

CCT was found to be highest in the pediatric age group. In adults, age and sex had no significant effect on this parameter. In the pediatric group, CCT was significantly higher in females. Studies in adults have reported no sex differences in CCT [30]. Limited pediatric studies have similarly reported no significant differences between sexes [19,20]. However, no study directly comparing pediatric and adult groups has been identified in the literature.

We believe that our study will contribute to the literature, particularly to the relatively scarce pediatric keratometry studies, by providing comparisons between sexes as well as between pediatric and adult groups. Given that normative data in these age and sex groups will gain importance with the increasing use of advanced imaging technologies, the relevance of our findings is evident.

This study has some limitations. One of them is that, although the study was conducted in a tertiary ophthalmology center, it was limited to the Turkish population. This limitation may serve as a guide for future studies focusing on ethnic differences. Another limitation is the exclusion of the preschool age group in the pediatric cohort due to poor cooperation during imaging. The retrospective design and relatively small sample size are also limitations. Although all measurements were performed using the same device and protocol at a single tertiary center, the retrospective nature of the data may limit full standardization and generalizability of the findings. All imaging was performed under mesopic conditions as part of the standard departmental protocol; however, given the retrospective design, slight variations in ambient illumination cannot be completely ruled out. Another limitation is the absence of an intra- or inter-observer reproducibility analysis. Although previous validation studies have demonstrated high reproducibility of the Anterion SS-OCT system, reporting reproducibility within each dataset would further strengthen normative data studies [34,35]. Future prospective work is planned to include this component. Furthermore, the single-center design does not allow for accurate generalizations to the entire population, restricting the findings to this center. Nevertheless, despite these limitations, our results are consistent with both small and large cohort studies in the literature. Another limitation is that it should be noted that there were slightly more female participants in the pediatric group, which may influence the results due to hormonal fluctuations during puberty. Future studies should consider the impact of sex-specific hormonal periods when analyzing pediatric ocular parameters.

The normative anterior segment data obtained in this study provide an important reference for both pediatric and adult populations. In the pediatric age group, particularly through the separate evaluation of preadolescent and adolescent periods, these findings may contribute to the early detection of deviations in ocular development. Such data can guide the management of clinical conditions, including pediatric keratoconus, accommodative strabismus, amblyopia, and secondary intraocular lens (IOL) planning following congenital cataract surgery. Furthermore, as normative data are quite limited for developmental cataract surgeries and the assessment of anterior segment development, the findings of this study serve as a valuable resource for clinical evaluation and decision-making processes.

In the adult population, these data can serve as a reference for interpreting preoperative measurements and monitoring changes following keratoconus progression or ocular surgery. Understanding age- and sex-related differences allows for individualized interventions and follow-up strategies in clinical practice, thereby enhancing patient safety and visual outcomes. Finally, this study provides an important reference for future research on pediatric and adult anterior segment parameters.

## 5. Conclusions

In this study, SS-OCT–derived anterior segment parameters—including keratometry, pachymetry, pupil diameter, WTW distance, and lens thickness—were systematically evaluated across different age groups and sexes. Our findings demonstrate that anterior and posterior corneal curvatures are steeper in females, while pupil diameter and lens thickness increase with age. In the pediatric population, corneal thickness was greater, and anterior as well as total astigmatism were higher, independent of sex. These normative data provide a valuable reference for the clinical assessment of corneal pathologies and precise biometric evaluations. Future research should focus on larger, more diverse pediatric cohorts to expand keratometric and biometric datasets, thereby enhancing clinical decision-making and surgical planning.

## Figures and Tables

**Table 1:** Comparison of anterior segment parameters among groups.

Parameters	Group 1 (*n* = 97)	Group 2 (*n* = 144)	Group 3 (*n* = 149)	*p*-Value	Pairwise *p*-Values		
					G1–G2	G2–G3	G1–G3
Mean ant Sim K (D)	43.80 ± 1.47	43.93 ± 1.70	43.71 ± 1.62	0.518 *	0.539 *	0.267 *	0.677 *
Sim K flat (D)	43.05 (42.15–44.26)	43.19 (42.21–44.52)	43.33 (42.19–44.34)	0.783 ^α^	0.558 ^α^	0.878 ^α^	0.520 ^α^
Sim K steep (D)	44.29 (43.28–45.59)	44.52 (43.27–45.92)	44.32 (43.29–45.33)	0.350 ^α^	0.555 ^α^	0.145 ^α^	0.498 ^α^
Ant steep astigmatism (D)	1.05 (0.74–1.67)	1.03 (0.58–1.71)	0.72 (0.42–1.15)	**0.021 ^α^**	0.982 ^α^	0.143 ^α^	**0.026 ^α^**
Kmax (D)	44.74 (43.90–46.42)	44.92 (43.55–46.51)	44.81 (43.66–46.07)	0.457 ^α^	0.950 ^α^	0.233 ^α^	0.373 ^α^
Mean post K (D)	−6.11 (−6.31–5.97)	−6.12 (−6.35–5.98)	−6.09 (−6.27–5.96)	0.318 ^α^	0.484 ^α^	0.133 ^α^	0.493 ^α^
Post K flat (D)	−5.96 (−6.16–5.80)	−5.99 (−6.18–5.83)	−5.97 (−6.11–5.84)	0.683 ^α^	0.510 ^α^	0.424 ^α^	0.982 ^α^
Post K steep (D)	−6.30 (−6.48–6.13)	−6.12 (−6.35–5.98)	−6.09 (−6.27–5.96)	0.112 ^α^	0.524 ^α^	0.052 ^α^	0.204 ^α^
Post Steep Astigmatism (D)	−0.30 (−0.41–0.24)	−0.32 (−0.45–0.23)	−0.29 (−0.36–0.20)	**0.017 ^α^**	0.986 ^α^	**0.023 ^α^**	0.108 ^α^
Post K max (D)	−6.39 (−6.60–6.22)	−6.39 (−6.69–6.20)	−6.37 (−6.56–6.20)	0.450 ^α^	0.753 ^α^	0.228 ^α^	0.402 ^α^
TCP mean (D)	43.39 ± 1.50	43.56 ± 1.75	43.31 ± 1.59	0.421 *	0.430 *	0.208 *	0.715 *
TCP flat (D)	42.79 ± 1.51	42.96 ± 1.60	42.84 ± 1.65	0.369 *	0.410 *	0.540 *	0.799 *
TCP steep (D)	43.74 (42.83–45.11)	44.08 (42.67–45.46)	43.83 (42.75–44.95)	0.493 ^α^	0.660 ^α^	0.224 ^α^	0.585 ^α^
Total steep astigmatism (D)	0.90 (0.59–1.33)	0.89 (0.50–1.54)	0.67 (0.38–1.13)	**0.007 ^α^**	0.942 ^α^	**0.024 ^α^**	**0.022 ^α^**
CCT (µm)	547.19 ± 36.5	534.73 ± 33.74	530.86 ± 33.85	0.001 *	0.017 *	0.603 *	0.001 *
TCT (µm)	541.86 ± 36	529.8 ± 32.65	525.03 ± 34.25	0.001 *	0.020 *	0.001 *	0.456 *
Pupil diameter (mm)	6.10 (5.40–6.70)	5.30 (4.70–6.0)	4.40 (3.90–5.0)	**<0.001 ^α^**	**<0.001 ^α^**	**<0.001 ^α^**	**<0.001 ^α^**
WTW distance (mm)	12.03(11.58–12.31)	11.83(11.56–12.14)	11.67 (11.42–11.98)	**<0.001 ^α^**	0.201 ^α^	**0.015 ^α^**	**<0.001 ^α^**
Lens thickness (mm)	3.47 (3.34–3.58)	3.84 (3.59–4.14)	4.39 (4.15–4.57)	**<0.001 ^α^**	**<0.001 ^α^**	**<0.001 ^α^**	**<0.001 ^α^**

* ANOVA, ^α^ Kruskal–Wallis test. CCT: central corneal thickness, TCT: thinnest point at corneal thickness, WTW: white to white, TCP: total corneal keratometric power, D: diopter, mm: millimeter, and µm: micrometer. Significant *p*-values are bold. A *p*-value < 0.05 was considered statistically significant. Data are presented as mean ± standard deviation for normally distributed variables and median (1st–3rd quartiles) for non-normally distributed variables.

**Table 2:** Effect size analysis of anterior segment parameters between groups.

Parameters	G1–G2 *p*	G1–G2 r	G1–G3 *p*	G1–G3 r	G2–G3 *p*	G2–G3 r
SimKmeanant (D)	0.539 *	0.09	0.677 *	0.09	0.267 *	0.11
SimKsteep (D)	0.555 **	0.038	0.498 **	0.043	0.145 **	0.085
SimKflat (D)	0.558 **	0.038	0.520 **	0.041	0.878 **	0.009
AstigSteep (D)	0.835 **	0.013	0.026 **	0.265 (G3 > G1)	<0.001 **	0.229 (G3 > G2)
Kmax (D)	0.950 **	0.004	0.373 **	0.057	0.233 **	0.070
postKmean (D)	0.484 **	0.045	0.493 **	0.044	0.133 **	0.088
postKsteep (D)	0.524 **	0.041	0.204 **	0.081	0.052 **	0.119
postKflat (D)	0.510 **	0.043	0.982 **	0.001	0.424 **	0.047
PostAstigSteep (D)	0.986 **	0.026	0.108 **	0.141	0.023 **	0.151 (G3 > G2)
PostKmax (D)	0.753 **	0.020	0.402 **	0.054	0.228 **	0.071
TCPmean (D)	0.430 *	0.09	0.715 *	0.08	0.208 *	0.12
TCPFlat (D)	0.410 *	0.07	0.799 *	0.02	0.540 *	0.03
TCPSteep (D)	0.660**	0.028	0.585 **	0.035	0.224 **	0.071
TCPastig (D)	0.942 **	0.014	0.022 **	0.178 (G3 > G1)	0.024 **	0.150 (G3 > G2)
CCT (µm)	0.017 *	0.33	0.001 *	0.67	0.603 *	0.62
TCT (µm)	0.020 *	0.32	0.456 *	0.68	0.001 *	0.62
PD (mm)	<0.001 **	0.324 (G2 > G1)	<0.001 **	0.617 (G3 > G1)	<0.001 **	0.413 (G3 > G2)
WTW (mm)	0.201 **	0.123	<0.001 **	0.269 (G3 > G1)	0.015 **	0.167 (G3 > G2)
LT (mm)	<0.001 **	0.521 (G2 > G1)	<0.001 **	0.798 (G3 > G1)	<0.001 **	0.616 (G3 > G2)

* ANOVA ** Kruskal–Wallis test. r: Effect size. For ANOVA, r values were converted from Cohen’s d; for Kruskal–Wallis, Rosenthal’s r was used. G1, G2, and G3 denote Group 1, Group 2, and Group 3, respectively. The expressions in parentheses indicate which group shows a higher value compared to the other. CCT: central corneal thickness, TCT: thinnest point at corneal thickness, WTW: white to white, TCP: total corneal keratometric power, PD: pupil diameter, LT: lens thickness, D: diopter, mm: millimeter, and µm: micrometer. Significant *p*-values are shown in bold. A *p*-value < 0.05 was considered statistically significant.

**Table 3:** Analysis of the pediatric group into adolescent and preadolescent age groups.

Parameters	Group 1 (*n* = 97)		*p*-Value
	Preadolescence (6–10 Years) (*n* = 21)	Adolescence (11–17 Years) (*n* = 76)	
Mean ant Sim K (D)	44.00 ± 1.61	43.75 ± 1.44	0.497 *
Sim K flat (D)	43.39 ± 1.73	43.11 ± 1.44	0.447 *
Sim K steep (D)	44.63 ± 1.57	44.42 ± 1.59	0.598 *
Ant steep astigmatism (D)	0.95 (0.71–1.62)	1.05 (0.75–1.69)	0.733 **
Kmax (D)	45.19 ± 1.71	44.7 ± 1.59	0.695 *
Mean post K (D)	−6.20 ± 0.23	−6.12 ± 0.21	0.511 *
Post K flat (D)	−6.07 ± 0.22	−5.95 ± 0.20	0.438 *
Post K steep (D)	−6.34 ± 0.24	−6.29 ± 0.24	0.626 *
Post Steep Astigmatism (D)	−0.26 (−0.34–0.22)	−0.31 (−0.44–0.24)	0.093 **
Post K max (D)	−6.41 ± 0.24	−6.38 ± 0.26	0.624 *
TCP mean (D)	43.68 ± 1.68	43.21 ± 1.43	0.474 *
TCP flat (D)	43.14 ± 1.70	42.72 ± 1.40	0.487 *
TCP steep (D)	44.23 ± 1.73	43.69 ± 1.51	0.507 *
Total steep astigmatism (D)	0.80(0.60–1.56)	0.91(0.58–1.26)	0.967 **
CCT (µm)	556.12 ± 40.67	541.68 ± 34.67	0.129 *
TCT (µm)	552.43 ± 39.81	537.96 ± 35.25	0.289 *
PD (mm)	6.0 ± 0.97	5.93 ± 0.86	0.973 *
WTW distance (mm)	11.91 (11.56–12.05)	12.09 (11.60–12.31)	0.110 **
LT (mm)	3.47 ± 0.20	3.48 ± 0.19	0.757 *

** Mann–Whitney U test, * *t*-test, CCT: central corneal thickness, TCT: thinnest point at corneal thickness, WTW: white to white, TCP: total corneal keratometric power, PD: pupil diameter, LT: lens thickness, D: diopter, mm: millimeter, and µm: micrometer. Data are presented as the mean ± standard deviation for normally distributed variables and median (1st–3rd quartiles) for non-normally distributed variables. A *p*-value < 0.05 was considered statistically significant.

**Table 4:** Sex-based comparisons within each group.

Parameters	Group 1 (*n* = 97)		*p*-Value	Group 2 (*n* = 144)		*p*-Value	Group 3 (*n* = 149)		*p*-Value
	Female	Male		Female	Male		Female	Male	
Mean ant Sim K (D)	44.03 ± 1.48	43.37 ± 1.37	0.034 *	44.13 ± 1.66	43.37 ± 1.37	0.031 *	43.99 ± 1.47	43.11 ± 1.78	0.002 *
Sim K flat (D)	43.30 (42.23–44.43)	42.37 (41.74–43.91)	0.534 ^†^	43.35 (42.50–44.74)	42.73 (41.99–43.80)	**0.012 ** ^†^	43.44 (42.41–44.42)	43.04 (41.64–43.96)	**0.007 ** ^†^
Sim K steep (D)	44.33 (43.28–45.56)	43.64 (42.95–45.04)	0.091 ^†^	44.67 (43.38–46.12)	43.93 (42.87–45.40)	0.129 ^†^	44.38 (43.17–45.47)	43.84 (42.32–44.96)	**0.033 ** ^†^
Ant steep astigmatism (D)	1.04 (0.71–1.51)	0.91 (0.71–1.40)	0.765 ^†^	1.03(0.60–1.42)	0.98 (0.45–1.80)	0.606 ^†^	0.71 (0.46–1.09)	0.70 (0.25–1.19)	0.520 ^†^
Kmax (D)	44.86 (43.77–46.12)	44.23 (43.33–45.48)	0.205 ^†^	45.07 (43.64–46.55)	44.64 (43.25–45.80)	0.073 ^†^	44.88 (43.55–46.19)	44.04 (42.70–45.69)	0.051 ^†^
Mean post K (D)	−6.13 (−6.32–6.0)	−6.05 (−6.28–5.88)	0.076 ^†^	−6.16 (−6.41v6.0)	−6.06 (−6.26–5.90)	**0.009 ** ^†^	−6.15 (−6.27–5.98)	−6.03 (−6.21–5.90)	**0.007 ** ^†^
Post K flat (D)	−5.98 (−6.18–5.83)	−5.88 (−6.02–5.76)	**0.045 ** ^†^	−6.03 (−6.20–5.86)	−5.90 (−6.07–5.74)	**0.003 ** ^†^	−5.99 (−6.12–5.86)	−5.87 (−6.07–5.72)	**0.004 ** ^†^
Post K steep (D)	−6.29 (−6.48–6.18)	−6.23 (−6.42–6.01)	0.109 ^†^	−6.33 (−6.60–6.15)	−6.21 (−6.45–6.03)	**0.032 ** ^†^	−6.27 (−6.43–6.10)	−6.21 (−6.35–6.02)	**0.036 ** ^†^
Post Steep Astigmatism (D)	−0.29 (−0.38–0.24)	−0.28 (−0.40–0.22)	0.634 ^†^	−0.32 (−0.42–0.24)	−0.29 (−0.45–0.20)	0.550 ^†^	−0.27 (−0.34–0.20)	−0.27 (−0.35–0.19)	0.846 ^†^
Post K max (D)	−6.38 (−6.59–6.25)	−6.31 (−6.49–6.11)	0.412 ^†^	−6.42 (−6.69–6.23)	−6.32 (−6.62–6.06)	**0.036 ** ^†^	−6.37 (−6.57–6.22)	−6.31 (−6.52–6.09)	0.096 ^†^
TCP mean (D)	43.62 ± 1.53	42.97 ± 1.37	0.042 *	43.72 ± 1.69	43.16 ± 1.85	0.081 *	43.55 ± 1.50	42.81 ± 1.67	0.008 *
TCP flat (D)	43.16 ± 1.56	42.47 ± 1.59	0.036 *	43.16 ± 1.56	42.47 ± 1.59	0.018 *	43.13 ± 1.50	42.22 ± 1.80	0.002 *
TCP steep (D)	43.78 (42.83–45.09)	43.13 (42.33–44.49)	0.150 ^†^	44.14 (42.72–45.53)	43.39 (42.49–45.0)	0.182 ^†^	43.89 (42.80–44.98)	43.31 (41.76–44.48)	0.091 ^†^
Total steep astigmatism (D)	0.91 (0.58–1.33)	0.76 (0.51–1.25)	0.893 ^†^	0.89 (0.52–1.27)	0.76 (0.43–1.74)	0.954 ^†^	0.66 (0.39–1.08)	0.59 (0.33–1.22)	0.912 ^†^
CCT (µm)	546.39 ± 35.18	548.67 ± 39.54	0.771 *	537.10 ± 31.97	528.97 ± 37.48	0.190 *	527.34 ± 31.14	538.51 ± 38.34	0.061 *
TCT (µm)	541.93 ± 34.33	541.73 ± 39.49	0.979 *	532.04 ± 29.43	524.35 ± 39.27	0.200 *	522.39 ± 31.60	531.40 ± 38.99	0.124 *
Pupil diameter (mm)	5.80 (5.20–6.35)	6.20 (5.57–6.62)	0.051 ^†^	5.30 (4.67–6.0)	5.30 (4.80–5.90)	0.876 ^†^	4.55 (4.0–5.07)	4.0 (3.55–4.70)	**0.004 ** ^†^
WTW distance (mm)	11.95 (11.50–12.25)	12.16 (11.92–12.38)	**0.036 ** ^†^	11.76 (11.47–12.11)	11.91 (11.61–12.33)	0.060 ^†^	11.57 (11.31–11.86)	12 (11.63–12.30)	**<0.001 ** ^†^
Lens thickness (mm)	3.50 (3.34–3.61)	3.42 (3.33–3.59)	0.108 ^†^	3.87 (3.63–4.24)	3.78 (3.56–4.03)	0.121 ^†^	4.36 (4.12–4.54)	4.47 (4.22–4.83)	**0.044 ** ^†^

^†^ Mann–Whitney U test, * *t*-test, CCT: central corneal thickness, TCT: thinnest point at corneal thickness, WTW: white to white, TCP: total corneal keratometric power, D: diopter, mm: millimeter, and µm: micrometer. Data are presented as the mean ± standard deviation for normally distributed variables and median (1st–3rd quartiles) for non-normally distributed variables. Significant *p*-values are shown in bold. A *p*-value < 0.05 was considered statistically significant.

**Table 5:** Effect size analysis of sex differences between groups.

Parameter	Male (*n* = 123) *p*	Male η^2^	Male r	Female (*n* = 267) *p*	Female η^2^	Female r
SimKmeanant (D)	0.597 *	0.0086	0.093	0.822 *	0.0015	0.039
SimKsteep (D)	0.669 **	0.0034	0.058	0.551 **	0.0030	0.055
SimKflat (D)	0.988 **	0.0004	0.020	0.872 **	0.0009	0.030
AstigSteep (D)	0.021 **	0.034	**0.184**	0.001 **	0.037	**0.192**
Kmax (D)	0.684 **	0.0027	0.052	0.572 **	0.0036	0.060
postKmean (D)	0.667 **	0.0035	0.059	0.413 **	0.0064	0.080
postKsteep (D)	0.628 **	0.0042	0.065	0.156 **	0.0133	0.115
postKflat (D)	0.882 **	0.0018	0.042	0.699 **	0.0056	0.075
PostAstigSteep (D)	0.420 **	0.0058	0.076	0.021 **	0.034	**0.184**
PostKmax (D)	0.680 **	0.0028	0.053	0.418 **	0.0103	0.102
TCP (D)	0.606 *	0.0083	0.091	0.723 *	0.0024	0.049
TCPFlat (D)	0.777 *	0.0042	0.065	0.855 *	0.0012	0.035
TCPSteep (D)	0.826 **	0.0019	0.044	0.649 **	0.0060	0.077
TCPastig (D)	0.317 **	0.0080	0.089	0.014 **	0.032	**0.179**
CCT (µm)	0.088 *	0.0397	0.199	0.001 *	**0.0249**	0.158
TCT (µm)	0.162 *	0.0150	0.122	<0.001 *	**0.0284**	0.169
PD (mm)	<0.001 **	0.084	**0.290**	<0.001 **	0.070	**0.265**
WTW (mm)	0.276 **	0.011	0.105	<0.001 **	0.025	**0.158**
LT (mm)	<0.001 **	0.093	**0.305**	<0.001 **	0.105	**0.324**

* ANOVA test, ** Kruskal–Wallis test. ANOVA effect size calculated as η^2^, Kruskal–Wallis test with Rosenthal’s r used for effect size. CCT: central corneal thickness, TCT: thinnest point at corneal thickness, WTW: white to white, TCP: total corneal keratometric power, PD: pupil diameter, LT: lens thickness, D: diopter, mm: millimeter, and µm: micrometer. Significant *p*-values are shown in bold. A *p*-value < 0.05 was considered statistically significant.

## Data Availability

The datasets generated during and/or analyzed during the current study are available from the corresponding upon on reasonable request.
